# Unveiling the TrkA-p35/CDK5 axis: a novel therapeutic target in diabetic kidney disease

**DOI:** 10.3389/fendo.2026.1791283

**Published:** 2026-03-12

**Authors:** Wenbo Xia, Mei Wang, Yongcai Gao, Yonghua Liu, Jing Li, Hongliang Zhang, Hongyan Luo, Dongyang Shen, Jing E, Bo Li, Yali Zheng

**Affiliations:** 1 People’s Hospital of Ningxia Hui Autonomous Region, Ningxia Medical University, Yinchuan, China; 2 Yinchuan Maternal and Child Health Care Hospital, Ningxia Medical University, Yinchuan, China

**Keywords:** TrkA, CDK5, p35, diabetic kidney disease, podocyte

## Abstract

**Objective:**

To investigate the potential crosstalk between TrkA, the high-affinity nerve growth factor receptor (NGFR), and cyclin-dependent kinase 5 (CDK5) in the pathogenesis of diabetic kidney disease (DKD). Furthermore, this study aims to evaluate the therapeutic potential of targeting TrkA in DKD.

**Methods:**

The renal transcriptional profiles were evaluated in db/db mice and controls. High glucose (HG) stimulation was used to induce an *in vitro* model of podocyte injury. The therapeutic effects of the TrkA inhibitor GW441756 were evaluated in both DKD model mice and HG-stimulated podocytes.

**Results:**

RNA sequencing detected NGFR upregulation in db/db mice. Phosphorylation of TrkA (Tyr490) increased in HG-stimulated podocytes, and TrkA overexpression aggravated HG-induced injury. Mechanistically, TrkA activation functionally links to CDK5 in the pathogenesis of DKD. Specifically, phosphorylation of TrkA at Tyr490 triggers the activation of the downstream ERK/EGR1 pathway. The accumulation of p35 activated CDK5, resulting in an inflammation-mediated podocyte injury. The TrkA inhibitor reduced its phosphorylation and attenuated downstream inflammation.

**Conclusion:**

Our findings suggest a TrkA-p35/CDK5 axis contributes to podocyte inflammation and injury, connecting neurotrophic signalling and renal metabolic inflammation through a novel mechanism. This work indicates that TrkA represents a potential therapeutic target for DKD therapy.

## 1 Introduction

Diabetic kidney disease (DKD) is a primary reason for mortality and disability globally (1, 2), affecting 15% to 40% of persons with diabetes. (3). Despite the wide use of glycemic control, ACE inhibitors and ARBs (4, 5) along with the advent of SGLT2 inhibitors has significantly slowed the progression of the disease (6), a proportion of individuals still progress to end-stage renal disease (ESRD). The prevalent pathological characteristics of DKD include glomerular hypertrophy, podocyte injury, tubulointerstitial fibrosis, and ultimately glomerulosclerosis (7, 8).

In the initial stages of diabetes, metabolic alterations disturb renal hemodynamics and induce inflammation (9). Hyperglycemia-induced podocyte impairment is a main factor in renal impairment in DKD (10). Podocytes are critical for maintaining selective permeability as they are a crucial element of the glomerular filtration barrier. Recent studies focusing on podocyte biology and *in vivo* gene deletion have shown that podocyte pathology initiates proteinuria and glomerulosclerosis (11). Podocytes undergo severe pathological alterations, such as apoptosis, phenotypic transformation, cytoskeletal disassembly and excess extracellular matrix production, under hyperglycaemic stress. These modifications ultimately compromise barrier function and precipitate proteinuria (12, 13).

The p35/CDK5 axis is recognized as a critical mediator of podocyte injury (14, 15). Consistent with these findings, our research group demonstrated that CDK5 was dysregulated in diabetes and contributed to cellular damage. Importantly, we further implicated NGF as a factor in this pathogenic mechanism (16, 17). Nevertheless, the exact molecular mechanism through which NGF signaling activates and upregulates the p35/CDK5 complex in the diabetic podocytes is not well characterized. To identify specific receptor-mediated events linking NGF to CDK5 activation, we conducted RNA sequencing on kidney tissues from mice with type 2 diabetes (18).

Transcriptomic analysis has identified alterations in several neurotrophic pathways and the high-affinity NGF receptor TrkA has emerged as a promising therapeutic target. TrkA is a receptor tyrosine kinase that is upregulated during renal inflammation (19, 20). In neuronal cells, NGF activates the p35/CDK5 complex, which subsequently phosphorylates its downstream substrates (21). The existence of a similar kinase-receptor interaction network—potentially involving other Trk family members or different receptor tyrosine kinases that interact with CDK5—and how metabolic and pathological stress regulates this network to influence the fate of renal cells and organ function remains to be determined. To clarify the specific roles of CDK5 and TrkA in diabetic nephropathy, direct molecular biological and pathological studies on renal tissues are required. Further exploration of the mechanisms related to TrkA and CDK5 not only deepens our understanding of the neurodevelopmental and pathological basis but also provides a crucial molecular bridge that connects the metabolic disorders of diabetes with its multiple systemic complications, particularly neurological and renal disorders, thereby holding significant translational medical value.

## 2 Materials and methods

### 2.1 Ethics committee

The animal care and use committee of Ningxia Medical University (Protocol No. IACUC-H2025098) authorized the animal experiments.

### 2.2 Antibodies

Anti-phospho-TrkA (pTyr490) antibody (Cat. No. RA18018-100) was purchased from Origene (Rockville, MD, USA). Antibodies against TrkA (Cat. No. 2505), Cdk5 (Cat. No. 2506), and p35/25 (Cat. No. 2680) were purchased from Cell Signaling Technology (Danvers, MA, USA). Antibodies against ERK (Cat. No. sc-514302), phospho-ERK (Cat. No. sc-7383), and Synaptopodin (Cat. No. sc-515842) were purchased from Santa Cruz Biotechnology (Dallas, TX, USA). Antibodies against EGR1 (Cat. No. 55117-1-AP), rabbit IgG (Cat. No. 30000-0-AP), and mouse IgG (Cat. No. B900620) were purchased from Proteintech (Wuhan, Hubei, China). Antibodies against Nephrin (Cat. No. ab216341), TNF-α (Cat. No. ab183218), IL-1β (Cat. No. ab234437), IL-6 (Cat. No. ab290735), and cleaved caspase-3 (Cat. No. ab214430) were acquired from Abcam (Cambridge, MA, USA). The antibody against β-Tubulin (Cat. No. GB11017-100) was purchased from Servicebio (Wuhan, Hubei, China).

### 2.3 Animal experiments

Seven-week-old C57BLKS/J db/db mice and their db/m counterparts were acquired from Huachuang Xinnuo Pharmaceutical Technology Co., Ltd. (Jiangsu, China) and housed under SPF setting, featuring temperature controlled at 22 ± 2°C, and humidity at 50% ± 5%. The mice were allowed to acclimatize for 1-week and provided unrestricted access to water and food without any restrictions. The mice were then randomized into different treatment groups after measuring fasting glucose in the blood, creatinine in the serum, and albumin levels in urine. All experimental methods and data assessments were carried out with the identities hidden. To investigate the impact of the pharmacological inhibition of TrkA in mice, eight-week-old male db/db mice were separated into the four groups: Control, db/db, db/db + NC Inhibitor, and db/db + TrkA Inhibitor. Low-dose GW441756 (10 mg/kg) was solubilized in DMSO and administered through tail vein injections once every five days over a two-week duration. Subsequently, mice were euthanized after intraperitoneal administration of pentobarbital sodium (50 mg/kg). The kidneys were quickly harvested after collecting the blood samples. Furthermore, 24-hour urine collections were conducted for biochemical examinations. One kidney was fixed in 4% paraformaldehyde solution and utilized for histological examinations. Using liquid nitrogen, the remaining kidney was quickly frozen and kept at -80 °C for subsequent protein and RNA extraction.

### 2.4 TrkA inhibition and overexpression in MPC5 cells

The MPC5 cell line is derived from renal podocytes and was acquired from Zhong Qiao Xin Zhou Biotechnology Co., Ltd. (Cat. No. ZQ-1119; Shanghai, China). MPC5 cells were cultured in DMEM (VivaCell) added with 10% FBS, 4 mM glutamine, and 1% P/S. The MPC5 cell line was serum-starved in low serum medium overnight before the test. The experiments were performed in media with low glucose (LG; 1 g/L) or high glucose (HG; 4.5 g/L).

MPC5 cells were cultured in DMEM with 10% FBS at 37 °C and 5% CO2. To establish stable lines, MPC5 cells at 85% confluence were trypsinized, resuspended in DMEM without serum at a concentration of 1 × 10^5^ cells/mL. A 6-well plate was inoculated with 1 mL of this suspension into each well. MPC5 cells were transduced with lentiviral particles encoding the TrkA overexpression vector or an empty control (Shanghai GeneChem Co., Ltd.). Stable cell lines were established via puromycin (2 µg/mL) selection for 72 h. For inhibitor studies, MPC5 cells underwent treatment with a TrkA inhibitor GW441756 (HY-18314, MedChemExpress, CA, USA).GW441756 was first dissolved in DMSO. We weighed 1 mg of GW441756 (MW 275.3) and dissolved it in 3.6 mL of DMSO to prepare a 1 mM stock solution. For cell treatment, 6 µL of the 1 mM stock was diluted into 6 mL of DMEM medium, resulting in a final GW441756 concentration of 1 µM and a final DMSO concentration of 0.1% (v/v). The concentration (1 µM) was selected based on CCK-8 cell viability assays, which confirmed no cytotoxicity at this dose (**Supplementary Figure 1A**), as well as on established literature (22). The ERK pathway inhibitor PD98059 (HY-12028, MedChemExpress, CA, USA). PD98059 was purchased as a 10 mM stock solution in DMSO. For cell treatment, 6 µL of the 10 mM stock was diluted into 6 mL of DMEM medium, resulting in a final PD98059 concentration of 10 µM and a final DMSO concentration of 0.1% (v/v). Our CCK-8 assays confirmed that 10 µM PD98059 (containing 0.1% DMSO) did not affect cell viability. This finding is consistent with established literature demonstrating no cytotoxicity at this dose (**Supplementary Figure 1B**), as well as on established literature (23). The experimental groups were categorized as LG, HG+Vehicle (0.1% DMSO), LG + Man, HG + TrkA Inhibitor, HG + empty vector, HG + TrkA overexpression vector, and HG + TrkA overexpression vector + PD98059.

We utilized small interfering RNA (siRNA) to specifically knock down TrkA expression in MPC5 cells. Concurrently, a rescue experiment was conducted by administering the TrkA inhibitor GW441756 to the TrkA overexpression group. The experimental groups were categorized as follows: LG, HG + si-NC, HG + si-TrkA, HG + TrkA overexpression control, and HG + TrkA overexpression + GW441756.

### 2.5 Biochemical analyses of urine and serum samples

Creatinine levels in both serum and urine were assessed using sarcosine oxidase in an automatic biochemical analyzer (Model: LST 008a) with reagents supplied by Maccura (Lot No.: 0125011). Urinary protein levels were estimated by immunoturbidimetry with reagents from Maccura (Lot No.: 0425021) as per the manufacturer’s guidelines.

### 2.6 RNA sequencing

We first utilized the Epicentre Ribo-Zero™ rRNA Removal Kit (Epicentre, WI, USA) to eliminate rRNA. Then, the NEB Next^®^ Ultra™ RNA Library Prep Kit (NEB, Ipswich, MA, USA) was utilized to create mRNA libraries. PCR amplification and sequencing was performed based on the manufacturer’s protocol.The raw data were then processed on the BMK Cloud platform (http://www.biocloud.net/) and the low-quality reads were discarded and the high quality reads were subsequently analyzed. Differential expression analysis was performed using the DESeq2 R package. Genes with an absolute log2 fold change (|log2FC|) ≥ 1 and an adjusted P-value (padj) < 0.05 were identified as differentially expressed genes (DEGs).The raw gene expression counts for all samples are provided in **Supplementary Table 1**. The complete differential expression analysis results are listed in **Supplementary Table 2**.

### 2.7 Alpha Fold 3 modeling

Sequences of amino acids for TrkA and CDK5 were downloaded from the UniProt database (https://www.uniprot.org/) and uploaded to the AlphaFold3 server (https://deepmind.google/technologies/alphafold/alphafold-server/) to generate models for predicting protein-protein interactions. Among the five models generated, Model 0 achieved the best evaluation result and was selected for in-depth inspection of the expected interaction interface. PyMOL 2.6, an open-source software, was utilized to display the amino acids participating in the interaction (24). These computational predictions only offer a structural hypothesis for the TrkA-CDK5 interaction and need to be validated in the future using biochemical methods.

### 2.8 Histological analysis

The kidney tissues that were removed from the mice were placed in a solution of four percent paraformaldehyde in order to be preserved, and they were then embedded in paraffin, sliced into sections 5 micrometers thick, and stained with H&E (G1120, Solarbio, Beijing, China). Alterations in renal histology were assessed using PAS and PASM staining techniques. PAS staining (C0142S, Beyotime, Shanghai, China) was used to evaluate matrix expansion within the glomeruli. PASM staining was used to evaluate the glomerular basement membrane using a PASM staining kit (DG0090, Leagene, Guangzhou, China).

### 2.9 Transmission electron microscopy

Renal cortex samples (<1 mm²) were fixed in 2.5% glutaraldehyde, rinsed with PBS, and post-fixed in 1% osmium tetroxide for TEM. The samples were then dehydrated using an ethanol series and embedded in Epon 812 resin for ultrathin sectioning. Images were acquired at 3,000 × magnification using a Hitachi HT7800 transmission electron microscope that was operated at a voltage of 80 kilovolts.

### 2.10 Immunofluorescence analysis

MPC5 cells were pretreated with the TrkA inhibitor GW441756 (1 µM), the ERK inhibitor PD98059 (10 µM), or a vehicle control for 24 h. Cells underwent fixation. The cells were blocked afterward with goat serum (Beyotime Biotechnology) for 40 minutes. Subsequently, the cells were left overnight at 4 °C with primary antibodies targeting p-TrkA, synaptopodin or p35. Next day, the cells were incubated with a fluorescent secondary antibody, at 37 °C for 60 minutes, and then stained with DAPI (D21490, Thermo Fisher Scientific). The Olympus fluorescence microscope (Tokyo, Japan) was used to acquire the images.

### 2.11 Western blotting

Total protein extracts were prepared from cultured cells and renal tissues using RIPA buffer (P0013B, Beyotime, Shanghai, China) with PMSF (SW106-01, Seven Bioltech, Beijing, China). Protein levels were assessed via the BCA assay. After SDS-PAGE separation and transfer to PVDF membranes, proteins were blocked with 5% BSA (SW127-02, Seven, Beijing, China).Then, membranes were incubated with primary antibodies overnight at 4 °C, followed by HRP-linked secondary antibodies (Affinity, China) for one hour. The ECL system served to reveal protein bands.We used the ImageJ software (Bethesda, MD, USA) to quantify band intensity.

### 2.12 RNA isolation and qRT-PCR

RNA extraction was performed on both cells and kidney tissues using the MolPure^®^ Flash Cell/Tissue Total RNA Kit (19221ES60, Yeasen, Gansu, China). After quantification, 1 µg RNA sample was reverse transcribed into cDNA with the PrimeScript RT Master Mix (RR036A, Takara, Gansu, China). Quantitative real-time PCR was performed using the QuantStudio™ 5 System (Thermo Fisher Scientific, CA, USA) with TB Green^®^ Premix Ex Taq™ II (RR820A, Takara, Gansu, China). Using the 2-ΔΔCt method, the target gene’s expression was calibrated with the housekeeping gene β-actin. The qPCR primers had the following sequences:

TNFα-F: 5′-atggcctccctctcatcagt-3′; TNFα-R: 5′-aaggtacaacccatcggctg-3′;
IL-6-F: 5′-gccttcttgggactgatgct-3′; IL-6-R: 5′-agcctccgacttgtgaagtg-3′;
IL-1β-F: 5′- gggctgcttccaaacctttg-3′; IL-1β-R: 5′-aagacacaggtagctgccac-3′;
βactin-F: 5′-cttcgttgccggtccacaccc-3′, βactin-R: 5′-ctgggcctcgtcacccacat-3′.

### 2.13 Flow cytometry

The evaluation of apoptosis was conducted using the Annexin V-FITC/PI kit (K240810, KeyGen Biotech, Jiangsu, China). Podocytes (5 × 10^5) were washed with PBS and resuspended in 500 μl of binding buffer. The samples were incubated in the dark at room temperature for 15 minutes following the addition of 5 μl of Annexin V-FITC and 5 μl of PI. We utilized a FACS Calibur Flow Cytometer (BD Biosciences) for cell analysis and processed the data with FlowJo v10.

### 2.14 Statistical data analysis

For the analysis of all statistical data, GraphPad Prism 8.0 was utilized. The findings are presented as mean ± standard deviation. For the analysis of data, One-way ANOVA was utilized for comparing multiple groups, followed by Bonferroni correction.

## 3 Results

### 3.1 Identification of NGFR as a potential upstream regulator in podocyte injury

**Figure 1:**
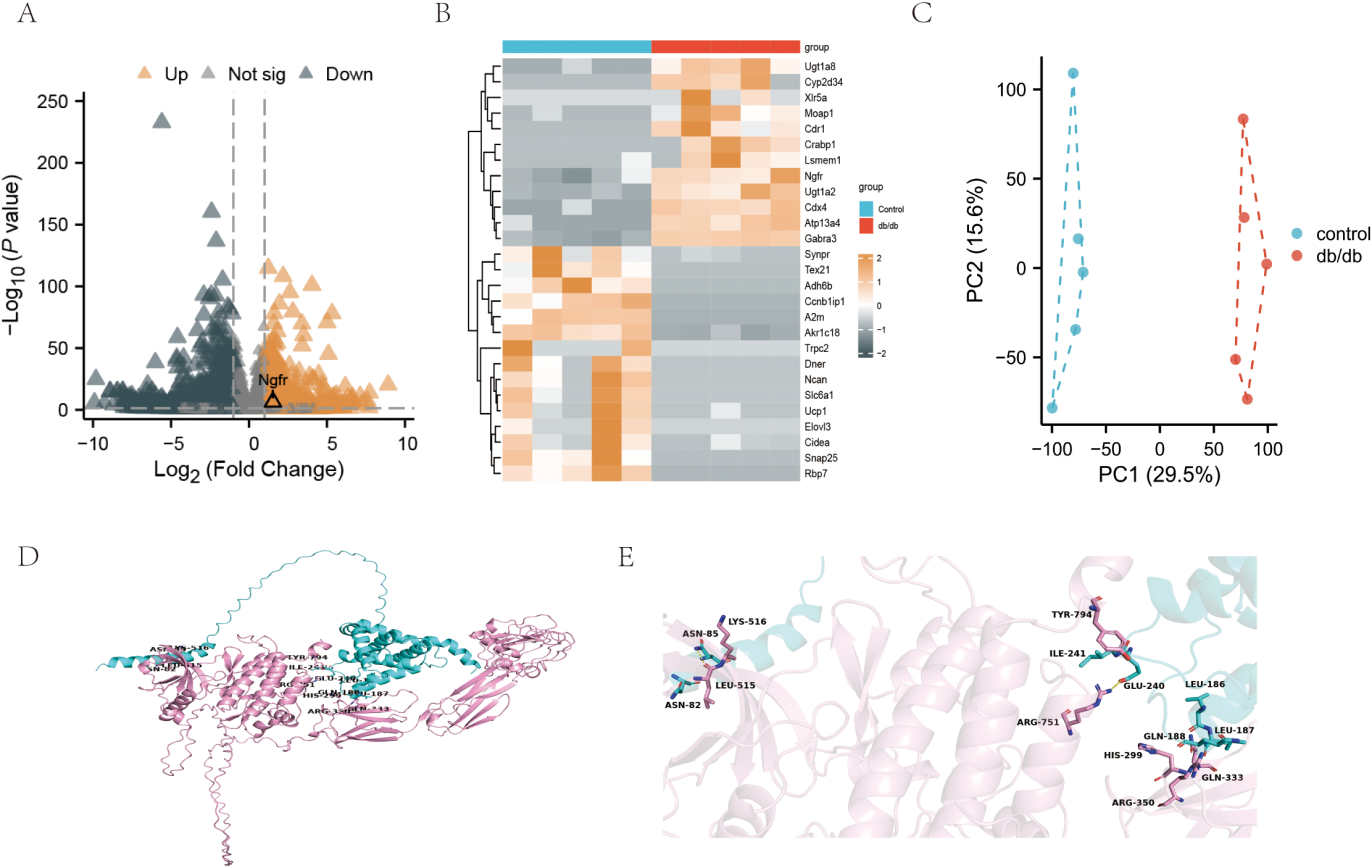
Identification of NGFR as a potential upstream regulator in podocyte injury. **(A)** Volcano plot of differentially expressed genes (|log2FC| ≥ 1; padj < 0.05) in renal tissues of db/db mice compared to controls. **(B)** Heatmap of gene expression profiles in renal tissues from control (n = 5) and db/db (n = 5) mice. Notably, NGFR expression was significantly upregulated (log2FoldChange = 1.53; padj = 4.75 × 10^-6^). **(C)** PCA score plot demonstrating the distinct separation between control and db/db groups. **(D, E)** AlphaFold3 modeling predicting the molecular interface between CDK5 and the kinase domain of TrkA.

RNA sequencing was conducted on renal tissues from db/db mice and controls to elucidate the molecular mechanisms underlying podocyte injury in DKD (18). The raw sequencing data and processed expression matrices are available in **Supplementary Table 1**. Our study revealed a significant upregulation of NGFR in db/db mice (**Figures 1A, B**). The PCA indicated that control and db/db mice showed statistically different expression spectrums (**Figure 1C**). Receptor tyrosine kinases, such as TrkA, frequently act as signal amplifiers, wherein even minor alterations in the receptor levels cause significant downstream effects. Consequently, TrkA was identified as a potential upstream regulator, prioritized over downstream effectors like Fscn1 which may represent passive responses to injury rather than initiating events. To investigate the structural basis for the crosstalk between TrkA and CDK5, we used AlphaFold3 to model the molecular interface. The structural predictions provided a structural hypothesis for this signaling axis, suggesting a potential molecular interface between CDK5 and the kinase domain of TrkA (**Figures 1D,E**). To functionally validate the TrkA-ERK-EGR1-p35/CDK5 signaling cascade, we assessed the phosphorylation of Tyr490 on TrkA, a critical site necessary for ERK pathway activation and subsequent CDK5 activation.

### 3.2 Hyperglycemia-induced TrkA phosphorylation is associated with podocyte inflammation and injury

**Figure 2:**
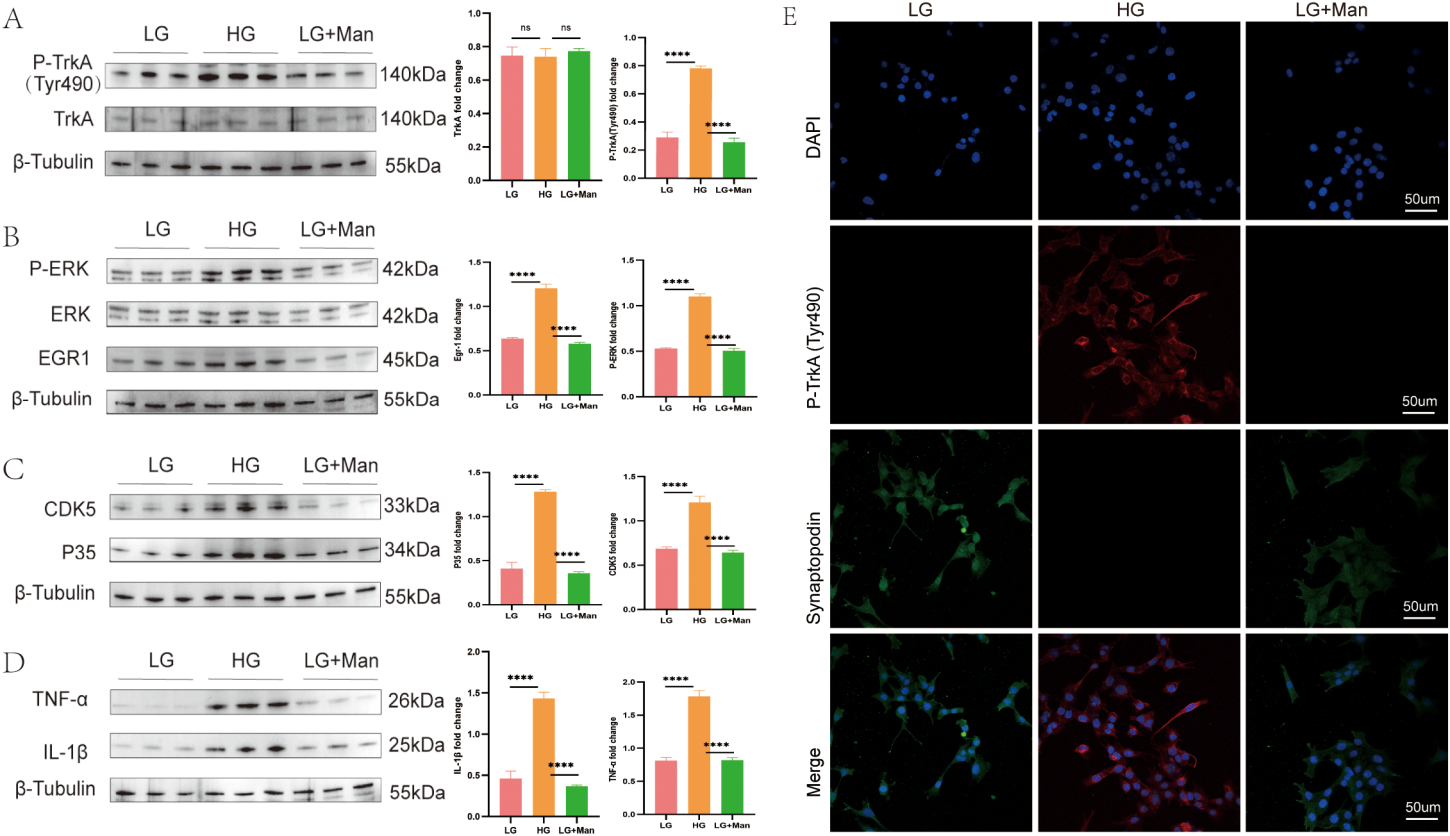
HG-induced TrkA phosphorylation is associated with podocyte inflammation and injury. **(A–D)** Representative western blots show the levels of **(A)** TrkA and phospho-TrkA (Tyr490); **(B)** ERK, phospho-ERK and EGR1; **(C)** p35 and CDK5; and **(D)** IL-1β and TNF-α in MPC5 cells from the LG, HG, and LG + Man groups at 24 h **(E)** Representative immunofluorescence double staining shows the expression of phospho-TrkA (Tyr490) and synaptopodin in MPC5 cells from the LG, HG, and LG + Man groups. ****p < 0.0001. p < 0.05 was considered statistically significant; ns indicates not significant.

To confirm the previous findings and investigate the underlying mechanisms, we established a cellular model using MPC5 cells treated with LG, HG, or mannitol as an osmotic control. Western blot analysis revealed that HG treatment induced TrkA phosphorylation and activation (**Figure 2A**). IF staining showed that p-TrkA levels were significantly elevated in the HG group (**Figure 2E**). Phosphorylation of TrkA at Tyr490 indicates activation of the ERK signaling pathway. Consistent with this, we observed HG stimulation was associated with ERK phosphorylation and subsequent EGR1 upregulation in MPC5 cells (**Figure 2B****).** Given the role of the ERK-EGR1 axis in regulating p35, HG-treated MPC5 cells demonstrated significantly higher p35 and CDK5 protein levels than in the control (**Figure 2C**). This signaling activation was functionally linked to podocyte injury. The significant upregulation of pro-inflammatory cytokines TNF-α and IL-1β following 24 h of HG exposure (**Figure 2D**). To further correlate TrkA activation with podocyte injury, we conducted double immunofluorescence staining for p-TrkA and the podocyte marker synaptopodin. IF double staining results demonstrated that synaptopodin levels were notably reduced in the HG group, thereby indicating podocyte damage (**Figure 2E**).Taken together, our evidence indicates that TrkA phosphorylation is functionally associated with HG-induced podocyte injury.

### 3.3 TrkA inhibitor suppresses p-TrkA-mediated upregulation of ERK, EGR1, and p35 in hyperglycemia-stimulated podocytes

**Figure 3:**
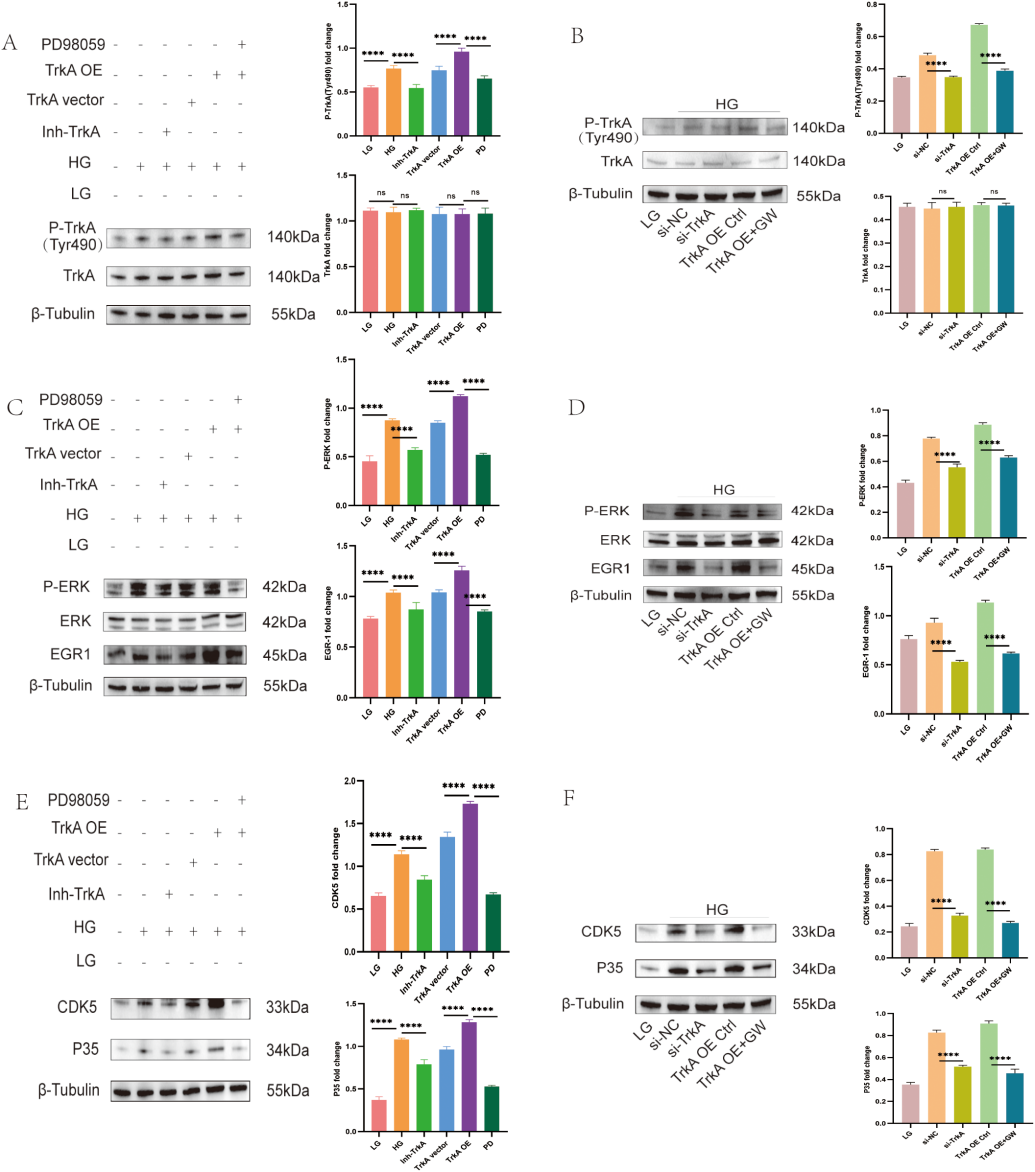
TrkA inhibitor suppresses p-TrkA-mediated upregulation of ERK, EGR1, and p35 in the MPC5 cells grown under HG conditions. Representative western blots show the levels of **(A)** TrkA and phospho-TrkA (Tyr490), **(C)** ERK, phospho-ERK, and EGR1, and **(E)** CDK5 and p35 in the LG, HG+Vehicle (0.1% DMSO), HG + Inh-TrkA, HG + empty vector, HG + TrkA OE, and HG + TrkA OE + PD98059 groups. Representative western blots show the levels of **(B)** TrkA and phospho-TrkA (Tyr490), **(D)** ERK, phospho-ERK, and EGR1, and **(F)** CDK5 and p35 in the LG, HG+si-NC, HG+si-TrkA, HG + TrkA OE+Ctrl, and HG + TrkA OE + GW441756 groups. *p < 0.05, **p < 0.01, ***p < 0.001, and ****p < 0.0001. p < 0.05 was considered statistically significant.

To investigate the signaling mechanism underlying TrkA-induced podocyte injury, we manipulated TrkA activity in the HG-treated MPC5 cells using the TrkA inhibitor GW441756 and lentiviral TrkA overexpression approaches. Treatment with GW441756 significantly reduced the levels of phosphorylated TrkA (**Figure 3A**). In the si-TrkA group, MPC5 cells exhibited a significant decrease in phosphorylated TrkA expression compared to the si-NC group (**Figure 3B**). Furthermore, the levels of phosphorylated TrkA were notably lower in the TrkA overexpression plus GW441756(TrkA OE+GW) group compared to the TrkA overexpression control (TrkA OE Ctrl) group (**Figure 3B**).

We subsequently analyzed the downstream effects of the ERK signaling pathway. Western blot analysis demonstrated that the levels of phosphorylated ERK (p-ERK) and EGR1 were significantly decreased in the Inh-TrkA group compared to the high glucose (HG) group (**Figure 3C**). In contrast, p-ERK and EGR1 levels were elevated in MPC5 cells transduced with TrkA-overexpressing lentivirus (TrkA OE) relative to those transduced with the empty vector (TrkA vector). Furthermore, p-ERK and EGR1 protein levels were diminished in the si-TrkA group compared to the si-NC control (**Figure 3D**). Similarly, p-ERK and EGR1 expression was reduced in the TrkA OE+GW group compared to the TrkA OE Ctrl group (**Figure 3D**).

We also analyzed the expression levels of CDK5 and its activator, p35. Compared to the HG group, the Inh-TrkA group exhibited significantly reduced levels of p35 and CDK5 proteins. In contrast, the TrkA OE group demonstrated elevated levels of p35 and CDK5 compared to the TrkA vector group (**Figure 3E**). The si-TrkA group showed lower expression of CDK5 and p35 proteins compared to the si-NC group (**Figure 3F**). Notably, the TrkA OE + GW group displayed decreased levels of CDK5 and p35 proteins relative to the TrkA OE Ctrl group (**Figure 3F**).

To determine whether ERK functions as the critical downstream mediator of TrkA in regulating CDK5, TrkA-overexpressing podocytes were treated with the ERK inhibitor PD98059. Pharmacological inhibition of ERK mitigated the effects of TrkA overexpression, with the TrkA OE + PD98059 group demonstrating significantly lower protein expression levels of p35 and CDK5 relative to the TrkA OE group alone (**Figure 3E**). The outcomes indicate that TrkA may enhance the inflammatory response of podocytes via the activation of ERK signaling pathways.

### 3.4 TrkA inhibition alleviates inflammation and injury in HG-stimulated podocytes

**Figure 4:**
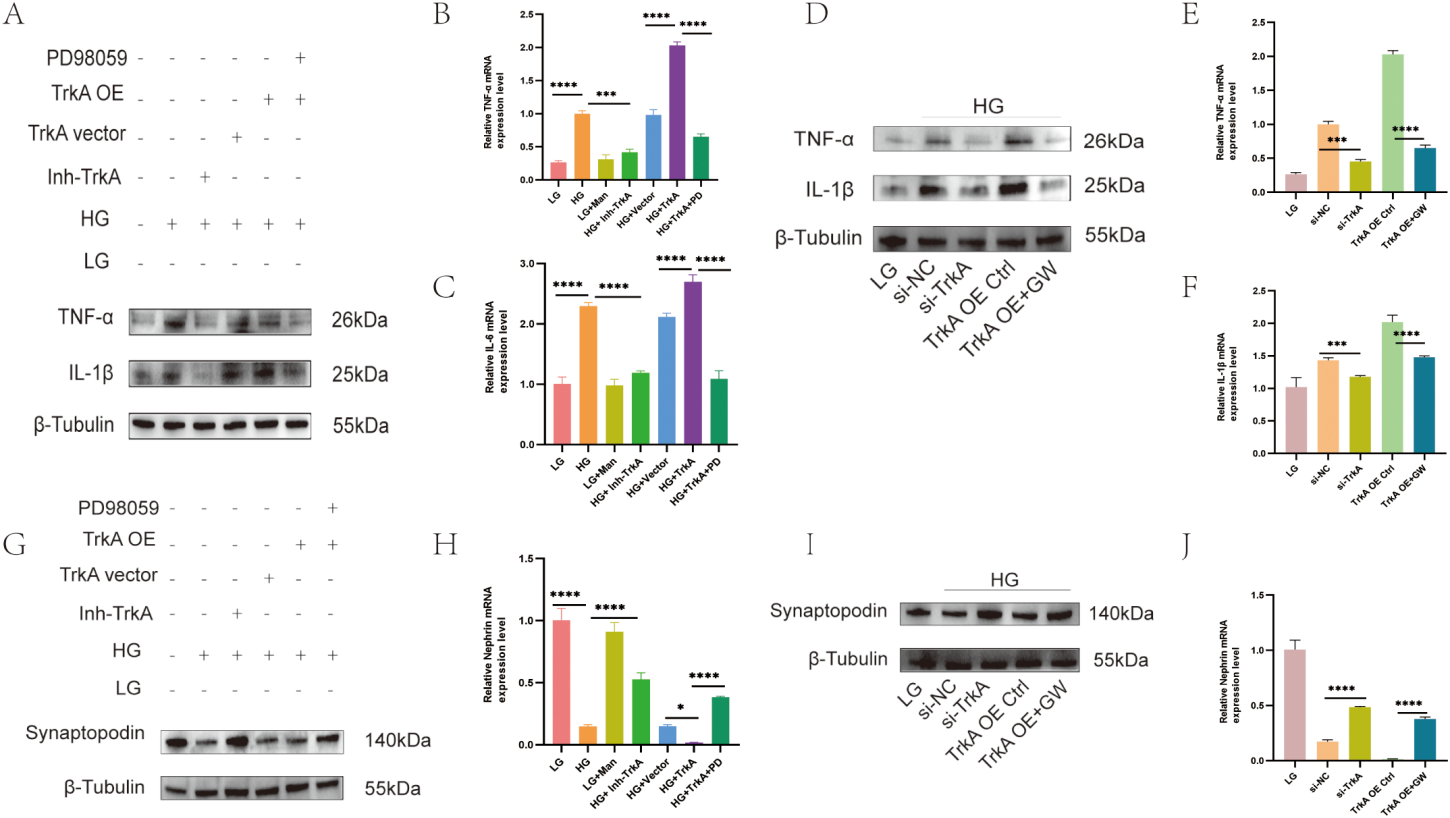
TrkA inhibition alleviates inflammation and injury in the HG-stimulated MPC5 cells. Representative western blots show the expression levels of **(A)** TNF-α and IL-1β, and **(G)** synaptopodin in the LG, HG+Vehicle, HG + Inh-TrkA, HG + empty vector, HG + TrkA OE, and HG + TrkA OE + PD98059 groups. Representative western blots show the expression levels of **(D)** TNF-α; IL-1β, and **(I)** synaptopodin in the LG, HG+si-NC, HG+si-TrkA, HG + TrkA OE+Ctrl, and HG + TrkA OE + GW441756 groups. Quantitative analysis shows the relative gene expression levels of **(B)** TNF-α; **(C)** IL-6; and **(H)** Nephrin in the LG, HG+Vehicle, LG + Man, HG + Inh-TrkA, HG + empty vector, HG + TrkA OE, and HG + TrkA OE + PD98059 groups. **(E)** TNF-α; **(F)** IL-1β; and **(J)** Nephrin in the LG, HG+si-NC, HG+si-TrkA, HG + TrkA OE+Ctrl, and HG + TrkA OE + GW441756 groups. *p < 0.05, **p < 0.01, ***p < 0.001, and ****p < 0.0001. p < 0.05 was considered statistically significant.

*In vitro*, we examined TrkA’s function by using GW441756 to inhibit its expression in MPC5 cells. Western blot analysis demonstrated that inhibition of TrkA significantly decreased the quantities of TNF-α and IL-1β in the HG-treated MPC5 cells. TrkA overexpression in MPC5 cells augmented HG-induced inflammatory markers in podocytes (**Figure 4A**). Additionally, inhibition of TrkA in the HG-treated MPC5 cells also reduced the expression levels of mRNA for pro-inflammatory mediators such as TNF-α and IL-6 (**Figures 4B, C**). Compared to the si-NC group, the si-TrkA group exhibited a significant reduction in the protein expression levels of TNF-α and IL-1β (**Figure 4D**). In contrast, MPC5 cells in the TrkA OE + GW group showed lower protein levels of TNF-α and IL-1β when compared to the TrkA OE Ctrl group (**Figure 4D**). Additionally, the mRNA levels of TNF-α and IL-1β were significantly downregulated in the TrkA OE + GW group relative to the TrkA OE Ctrl group (**Figures 4E,F**).

Western blot analysis also demonstrated that TrkA inhibition restored synaptopodin expression levels (**Figure 4G**) and nephrin mRNA levels after HG stimulation (**Figure 4H**). The si-TrkA group showed higher expression of synaptopodin and nephrin compared to the si-NC group (**Figures 4I, J**). The TrkA OE + GW group displayed increased levels of synaptopodin and nephrin relative to the TrkA OE Ctrl group (**Figures 4I, J**).

We used a TrkA-overexpressing podocyte cell line treated with ERK inhibitor PD98059. The TrkA OE group exhibited elevated expressions of TNF-α, IL-1β in podocytes than in the empty vector group (**Figure 4A**). MPC5 cells in the TrkA + PD98059 group demonstrated lower levels of TNF-α, IL-1β expression in comparison to the TrkA OE group (**Figure 4A**). This demonstrated that treatment with the ERK inhibitor effectively counteracted the pro-inflammatory effects of TrkA in the podocytes. The findings imply that TrkA enhances the inflammation reaction of podocytes by activating ERK signaling pathways.

### 3.5 The TrkA inhibitor alleviates apoptosis in HG stimulated podocytes

**Figure 5:**
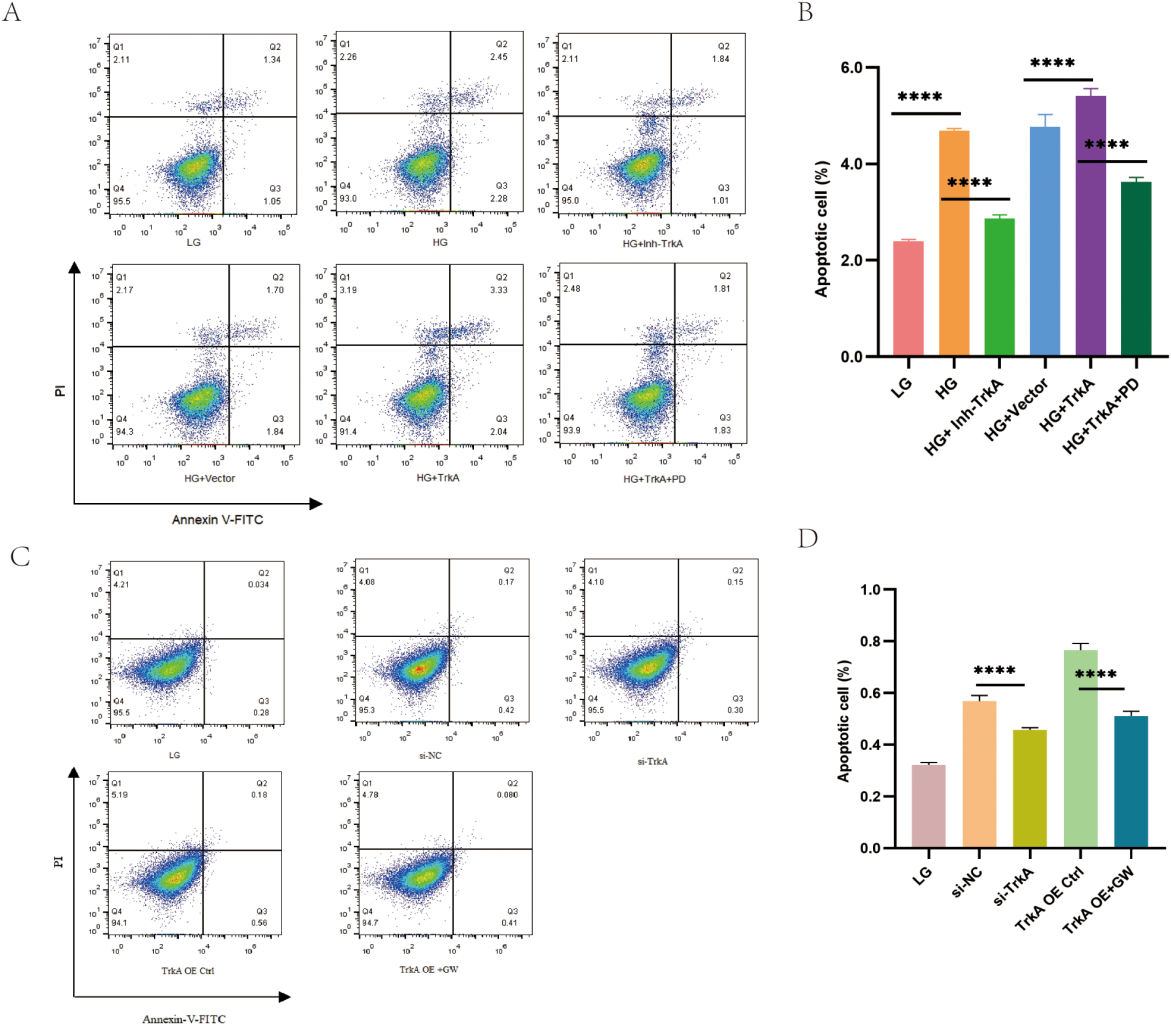
TrkA inhibition alleviates apoptosis in the HG-stimulated MPC5 cells. **(A)** Flow cytometry plots show the analysis of the percentage of apoptotic cells in the LG, HG+Vehicle, HG + Inh-TrkA, HG + empty vector, HG + TrkA OE, and HG + TrkA OE + PD98059 groups based on Annexin V/PI double staining. PI distinguishes viable cells from apoptotic cells; Annexin V-FITC specifically identifies apoptotic cells.**(B)** Quantitative analysis of the percent apoptotic cells in different groups based on data shown in **(A)**. **(C)** The analysis of the percentage of apoptotic cells in the LG, HG+si-NC, HG+si-TrkA, HG + TrkA OE+Ctrl, and HG + TrkA OE + GW441756 groups.**(D)** Quantitative analysis of the percent apoptotic cells in different groups based on data shown in **(C)**. *p < 0.05, **p < 0.01, ***p < 0.001, and ****p < 0.0001. p < 0.05 was considered statistically significant.

Flow cytometry analysis demonstrated that the TrkA inhibitor mitigated HG-induced podocyte apoptosis. In contrast, TrkA overexpression significantly sensitized MPC5 cells to HG-induced apoptosis (**Figure 5A**). MPC5 cells in the si-TrkA group exhibited reduced apoptosis compared to the si-NC group. Similarly, the TrkA OE + GW group showed lower apoptosis relative to the TrkA OE Ctrl group (**Figure 5C**). These data suggest that TrkA is critical for regulating podocyte apoptosis. Moreover, ERK inhibitor treatment reversed TrkA-induced podocyte apoptosis (**Figure 5A**). These results indicate that TrkA induces podocyte apoptosis through the activation of the ERK signaling cascade.

### 3.6 TrkA inhibition alleviates DKD in the db/db mice

**Figure 6:**
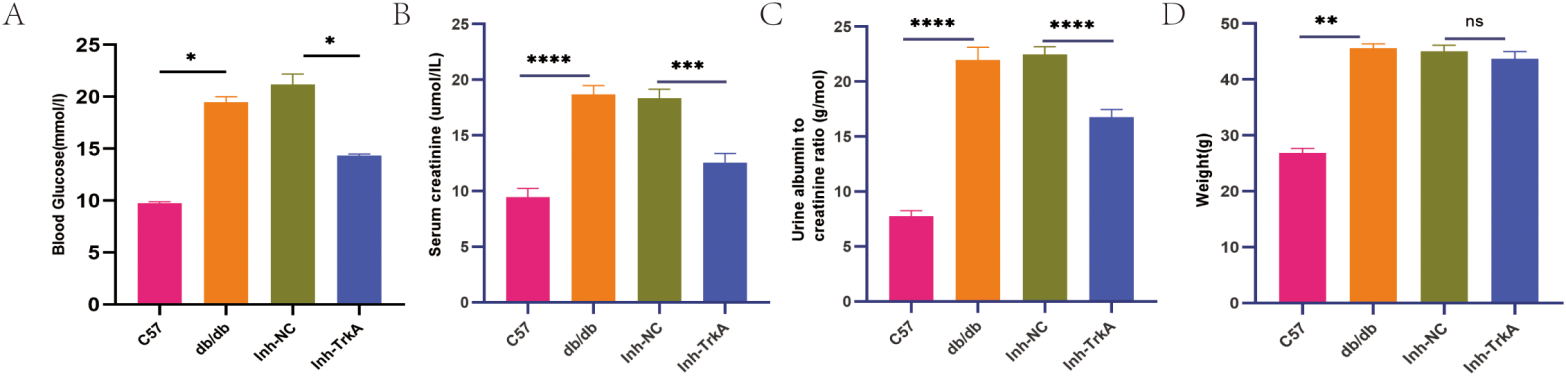
Inhibition of TrkA improves DKD in db/db mice. **(A)** Blood glucose levels; **(B)** Serum creatinine levels; **(C)** Urine albumin/creatinine ratio, and **(D)** weight in the control, db/db, Inh-NC, and Inh-TrkA groups of mice.*p < 0.05, **p < 0.01, ***p < 0.001, and ****p < 0.0001. p < 0.05 was considered statistically significant.

To examine the function of TrkA in DKD *in vivo*, we treated db/db mice with the TrkA inhibitor GW441756. Compared to controls, db/db mice showed severe impairment of the kidney as indicated by increased blood glucose (**Figure 6A**), serum creatinine (**Figure 6B**), and urinary albumin to creatinine ratio (**Figure 6C**). Notably, GW441756 treatment significantly reduced hyperglycemia (**Figure 6A**), suggesting a systemic metabolic effect of TrkA inhibition that represents a potential confounder. Nevertheless, TrkA inhibition significantly ameliorated kidney impairment, as shown by reduced serum creatinine levels and urinary albumin to creatinine ratio (**Figures 6B, C**). The db/db mice exhibited a higher body weight compared to the control mice; however, treatment with GW441756 did not result in significant changes in body weight when compared to the db/db mice (**Figure 6D**).

### 3.7 TrkA inhibition reduces the levels of p-TrkA, p-ERK, EGR1, p35, and CDK5 in renal tissues of db/db mice

**Figure 7:**
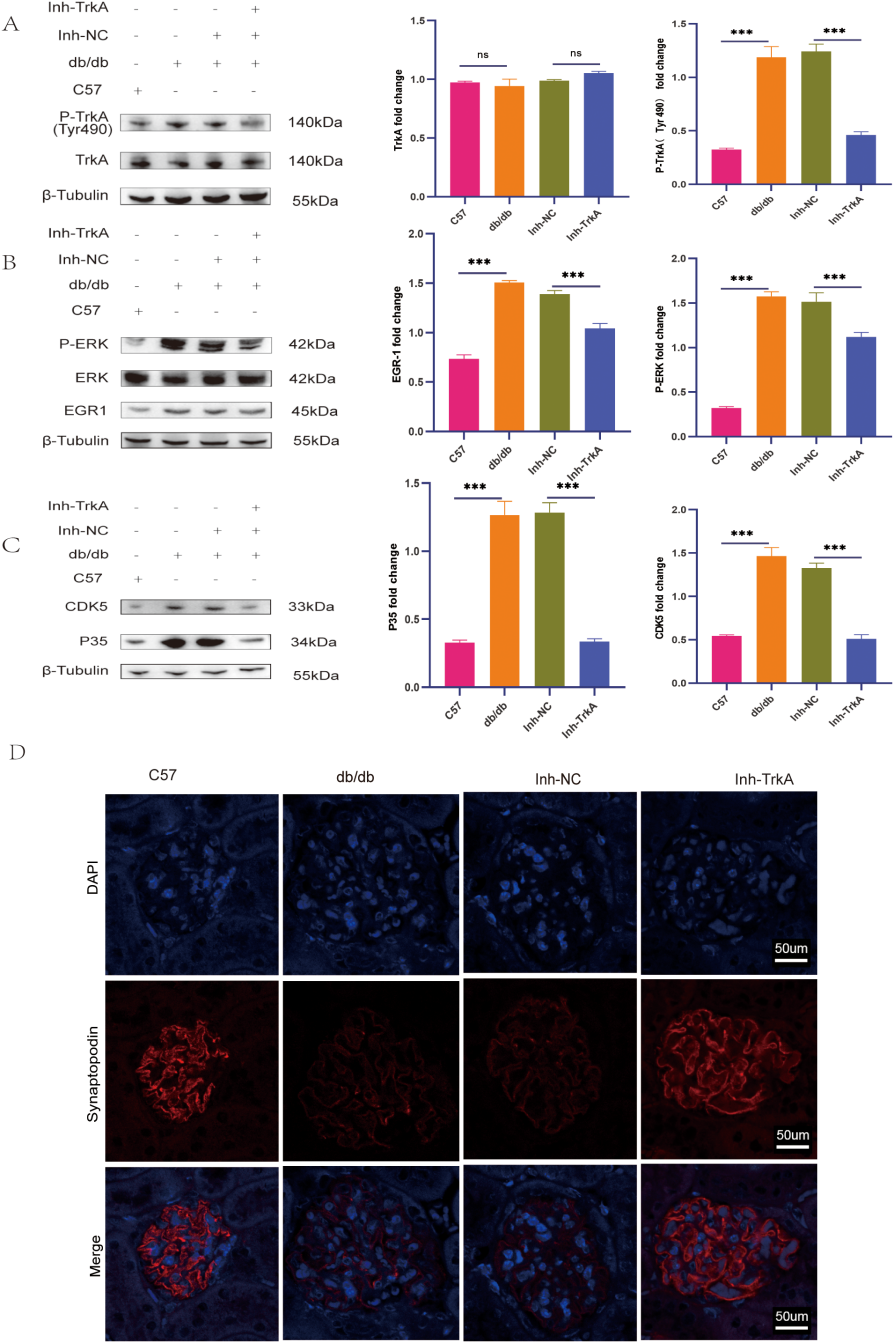
TrkA inhibition reduces the levels of p-TrkA, p-ERK, EGR1, p35, and CDK5 in the kidneys of db/db mice. **(A–C)** Representative western blots show the protein expression levels of **(A)** TrkA and phospho-TrkA (Tyr490), **(B)** ERK, p-ERK and EGR1, and **(C)** p35 and CDK5 in the kidney samples from the control, db/db, Inh-NC, and Inh-TrkA groups of mice. **(D)** Representative IF images show the expression of Synaptopodin (red) in the glomeruli of control, db/db, Inh-NC, and Inh-TrkA groups of mice (400X). ***p < 0.001. p < 0.05 was considered statistically significant; ns indicates not significant.

To elucidate the signaling pathways responsible for the pathological effects of TrkA in the db/db mice, we administered the TrkA inhibitor GW441756 to db/db mice. Western blot analysis revealed p-TrkA levels were elevated in db/db mice compared to controls, indicating pathway activation, but were significantly attenuated by GW441756 treatment (**Figure 7A**). Assessment of the downstream ERK pathway revealed that p-ERK and EGR1 levels were upregulated in db/db mice; however, TrkA inhibition effectively normalized these levels (**Figure 7B**). Furthermore, while p35 and CDK5 protein levels were increased in db/db mice relative to controls, their expression was downregulated in the Inh-TrkA group relative to the Inh-NC group (**Figure 7C**). To further correlate TrkA activation with podocyte injury in DKD, we conducted immunofluorescence staining for podocyte marker Synaptopodin. Consistent with a protective effect, the results indicated that TrkA inhibition increased Synaptopodin levels in db/db mice (**Figure 7D**). Collectively, these results imply that GW441756 ameliorates DKD by targeting the TrkA-ERK-EGR1-p35/CDK5 signaling axis in podocytes.

### 3.8 Inhibition of TrkA alleviates podocyte injury in type 2 diabetic mice

**Figure 8:**
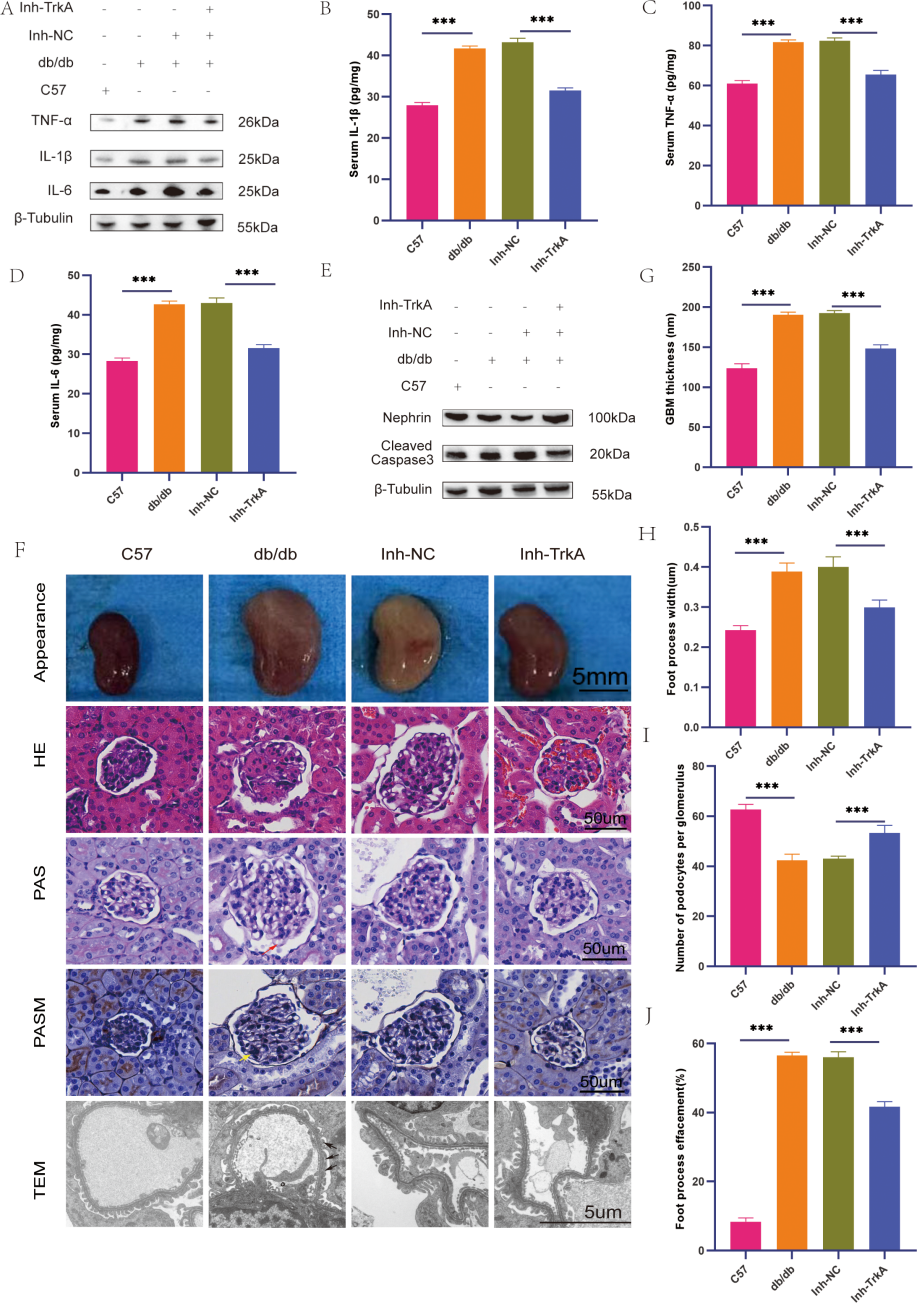
TrkA inhibition alleviates podocyte injury in the type 2 diabetic mice. **(A)** Representative Western blots showing the protein levels of IL-6, IL-1β, and TNF-α in renal tissues from control, db/db, db/db + NC inhibitor (Inh-NC), and db/db + TrkA inhibitor (Inh-TrkA) mice. **(B–D)** ELISA quantification of serum **(B)** IL-1β, **(C)** TNF-α, and **(D)** IL-6 levels in the four groups. **(E)** Representative western blots showing the protein levels of cleaved caspase-3 and Nephrin in each group. **(F)** Gross kidney appearance and histological analysis using H&E, PAS, and PASM staining of kidney tissues (400×). Representative transmission electron microscopy (TEM) images (3,000X) illustrating podocyte ultrastructure in each group. Images shown are representative of sections stained simultaneously under identical conditions. **(G–J)** Quantitative analysis of podocyte morphology: **(G)** Quantification of GBM thickness. **(H)** Measurement of foot process width. **(I)** Number of podocytes per glomerulus. **(J)** Assessment of foot process effacement. For TEM quantification, at least 15 glomeruli from 5 mice per group were analyzed. Data are presented as mean ± SD and were analyzed by one-way ANOVA followed by Bonferroni correction. ***p < 0.001. p < 0.05 was considered statistically significant.

To further investigate the function of TrkA in type 2 diabetes, we inhibited its activity in db/db mice. We found that TrkA inhibition significantly decreased the levels of inflammation-associated factors, including TNF-α, IL-1β, and IL-6 in the renal tissue of the db/db mice (**Figures 8A-D****).** Additionally, TrkA inhibition significantly decreased cellular apoptosis and reestablished the representation of podocyte markers, including nephrin, in the db/db mice (**Figure 8E**). Histological assessments confirmed that db/db mice showed advanced renal pathologies, such as: glomerular hypertrophy, expansion of the mesangial matrix, and capillary microaneurysms. Furthermore, electron microscopy demonstrated widespread effacement of podocyte foot processes in db/db mice. However, TrkA inhibition significantly alleviated mesangial expansion and pathological renal injury (**Figures 8F–J**).

## 4 Discussion

**Figure 9:**
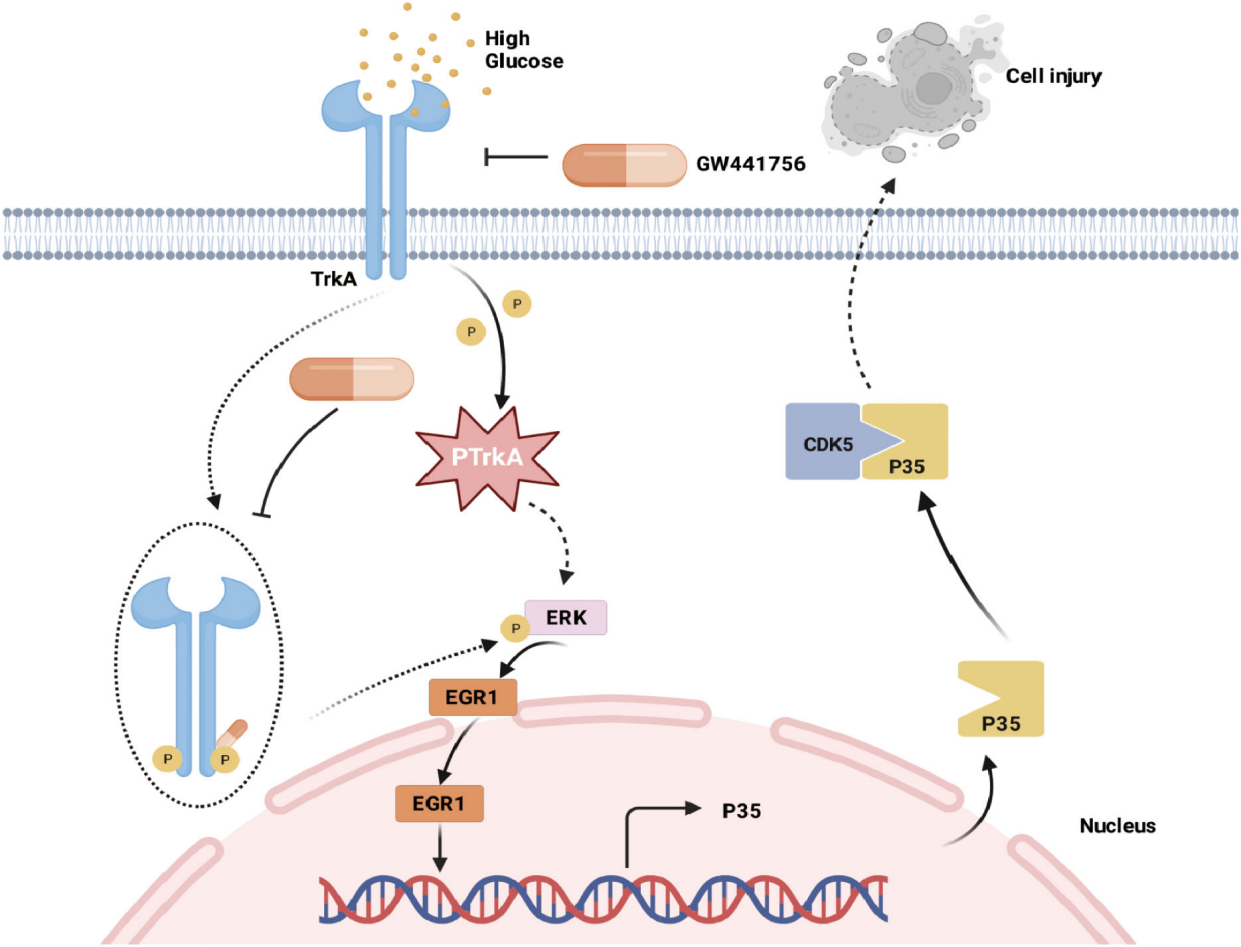
Schematic diagram shows the TrkA/ERK/p35/CDK5 regulatory axis and the site of action for the TrkA inhibitor GW441756 in the murine renal podocytes.

This research shows that upregulation and activation of TrkA triggers a specific signaling cascade involving ERK, EGR1, and p35/CDK5, leading to podocyte inflammation and injury in DKD. Furthermore, pharmacological inhibition of TrkA attenuates these pathological changes *in vitro* and *in vivo*. Schematic diagram is shown in **Figure 9**.

Podocyte injury alters glomerular filtration and is a primary cause of DKD (8, 25). Podocyte injury is critical for the development of proteinuria and is a component of the pathophysiology of DKD (26, 27). Unlike acute injuries, the primary pathological drivers in DKD are characterized by long-term, subtle gene regulatory changes, where accumulating evidence from preclinical and epidemiological studies increasingly implicates chronic inflammation (28, 29). Consequently, anti-inflammatory strategies that target specific intracellular signaling mechanisms have emerged as potential therapeutic avenues (30, 31). TrkA, a receptor tyrosine kinase, acts as a signal amplifier. Therefore, even minor increases in the TrkA gene expression lead to pronounced activation of the receptor itself (p-TrkA) and subsequent phosphorylation. Consequently, NGF receptor antagonists are currently being investigated as potential therapies for malignancies and pain management (32–36).The specific role of TrkA-mediated signaling in podocytes within DKD remains poorly understood. While individual components of the TrkA/ERK/CDK5 signaling axis have been studied in other contexts such as neuronal biology and oncology (21, 37), their specific interplay within the pathogenesis of DKD, particularly in podocytes, remains poorly understood. Our study introduces significant innovation by establishing a previously uncharacterized upstream regulatory mechanism.

Specifically, we demonstrate for the first time that TrkA acts as the critical upstream trigger linking hyperglycemic stress to the ERK-EGR1-p35/CDK5 axis in podocytes. Previous studies have focused on CDK5 activation via p25 in acute injuries (38); however, our data reveal a distinct pathogenic mechanism driven by long-term, subtle TrkA-mediated amplification of p35 under diabetic conditions. CDK5 governs critical aspects of the cell cycle (39). Its deregulation through the formation of p25 leads to pathological consequences (40) and affects immunity and metabolism (41–43). In the kidney, p35 is expressed in many podocytes and activates the Cdk5 kinase. Aberrant activation of the p35/CDK5 signaling pathway causes cytoskeletal disruption and inflammation in these cells (44). Previous research suggest that HG increases the levels and activity of CDK5 (45, 46). It is important to note that the proposed structural interaction between TrkA and CDK5 currently relies on AlphaFold3 computational predictions. Although our functional experiments support the existence of the TrkA-p35/CDK5 signaling axis, we did not conduct biochemical assays such as co-immunoprecipitation (Co-IP) or fluorescence resonance energy transfer (FRET). Thus, it remains to be established whether a direct physical interaction exists between these two proteins, necessitating further validation through structural biology methods in future studies.

Our research addresses a critical gap in understanding the upstream regulation of CDK5 by demonstrating that pharmacological inhibition of ERK with PD98059 reverses TrkA-induced CDK5 upregulation and subsequent cellular injury. This functionally validates CDK5 as the pivotal downstream effector of TrkA signaling in this context. The selection of the ERK-EGR1-p35 pathway is based on its well-established role in neurotrophin signaling and neuronal injury (21). We investigated whether this conserved signaling axis operates in renal podocytes under diabetic conditions. Our functional data supports this hypothesis, as ERK inhibition effectively attenuates hyperglycemia-induced p35 upregulation. Furthermore, considering that the ERK pathway mediates inflammatory responses (47), our study demonstrates that TrkA inhibition decreases ERK phosphorylation and reduces podocyte injury. However, a limitation of our study is that we did not definitively distinguish whether ERK regulates p35 primarily through EGR1-mediated transcription and/or via post-translational modulation of protein degradation. Given the complex regulation of p35, subsequent studies utilizing Chromatin Immunoprecipitation (ChIP) or protein stability assays (e.g., cycloheximide chase assays) are necessary to fully elucidate the intermediate mechanisms linking ERK activation to p35 accumulation in DKD.

The TrkA-p35/CDK5 pathway offers a new therapeutic target in DKD. This area integrates areas of neuroscience and nephrology through its ability to regulate survival and inflammatory pathways (48). Subsequent studies may establish whether the impairment of TrkA-p35/CDK5 in kidneys originates in the renal microenvironment or innervating neurons, and whether it is linked to fibrosis and functional decline (49, 50). Also, developing multi-target kinase inhibitors that can selectively inhibit the TrkA-p35/CDK5 axis in pathological circumstances is an important therapeutic strategy (51).

This study’s limitations should be noted. First, we did not include frontline drugs as positive controls, as our primary focus was on target validation rather than comparative efficacy. Although the pathogenic role of TrkA in DKD was confirmed, the efficacy of its inhibition was not directly compared with frontline clinical drugs such as ACEIs, ARBs, or SGLT2 inhibitors; thus, the clinical superiority of targeting this pathway requires further investigation. Second, although the kidney-to-body weight ratio was not calculated, the absence of weight loss suggests that renal protection was not due to a reduction in body mass, a conclusion further supported by histological improvements. Third, although the selected dose of GW441756 demonstrated safety and efficacy, and its specificity was validated through siRNA experiments, future preclinical studies are needed to determine the optimal therapeutic window. Furthermore, while the reduction in UACR and the restoration of slit diaphragm proteins suggest functional recovery, the absence of direct permeability assays or serial proteinuria kinetics limits the dynamic assessment of glomerular barrier restoration. Finally, as this study utilized an 8-week db/db mouse model, the efficacy of TrkA inhibition requires validation in more advanced nephropathy models, and long-term safety data necessary for clinical translation are currently lacking.

Elucidating these mechanisms clarifies the molecular crosstalk between diabetic metabolic dysregulation and neuro-renal pathology, thereby facilitating clinical translation. This study aligns with current research trends regarding multifactorial crosstalk and immunometabolic inflammation in DKD. Notably, emerging mechanisms such as diabetic tubulopathy (52), epigenetic regulation (53), gut microbiota metabolites, and programmed cell death (54) are increasingly recognized as critical components in the progression of DKD. Future investigations into the interplay between the TrkA-p35/CDK5 axis and these emerging pathways would further enhance our understanding of the systemic nature of DKD.

## 5 Conclusions

This study indicates that the TrkA-ERK-EGR1-p35/CDK5 axis influences podocyte inflammation and injury. TrkA is a key upstream receptor, and subtle variations in its expression levels can significantly influence DKD progression. By connecting neurogenic receptors with renal metabolic inflammation, our work provides a new explanatory dimension for DKD pathogenesis.

## Data Availability

The datasets presented in this study can be found in online repositories. The names of the repository/repositories and accession number(s) can be found in the article/**Supplementary Material**.

## Supplementary material

Supplementary Figure 1 Effect of different concentrations of GW441756 and PD98059 on cell viability.Cell viability was assessed using the CCK-8 assay following treatment with various concentrations of **(A)** GW441756 and **(B)** PD98059.

Supplementary Table 1 Raw gene expression counts for all samples.

Supplementary Table 2 Complete list of differentially expressed genes and statistical analysis results.
