# Supplementary figures and images for: Unveiling the TrkA-p35/CDK5 axis: a novel therapeutic target in diabetic kidney disease
Source: Front Endocrinol (Lausanne). 2026 Mar 12;17:1791283. doi: 10.3389/fendo.2026.1791283 (PMC13017383; doi:10.3389/fendo.2026.1791283)

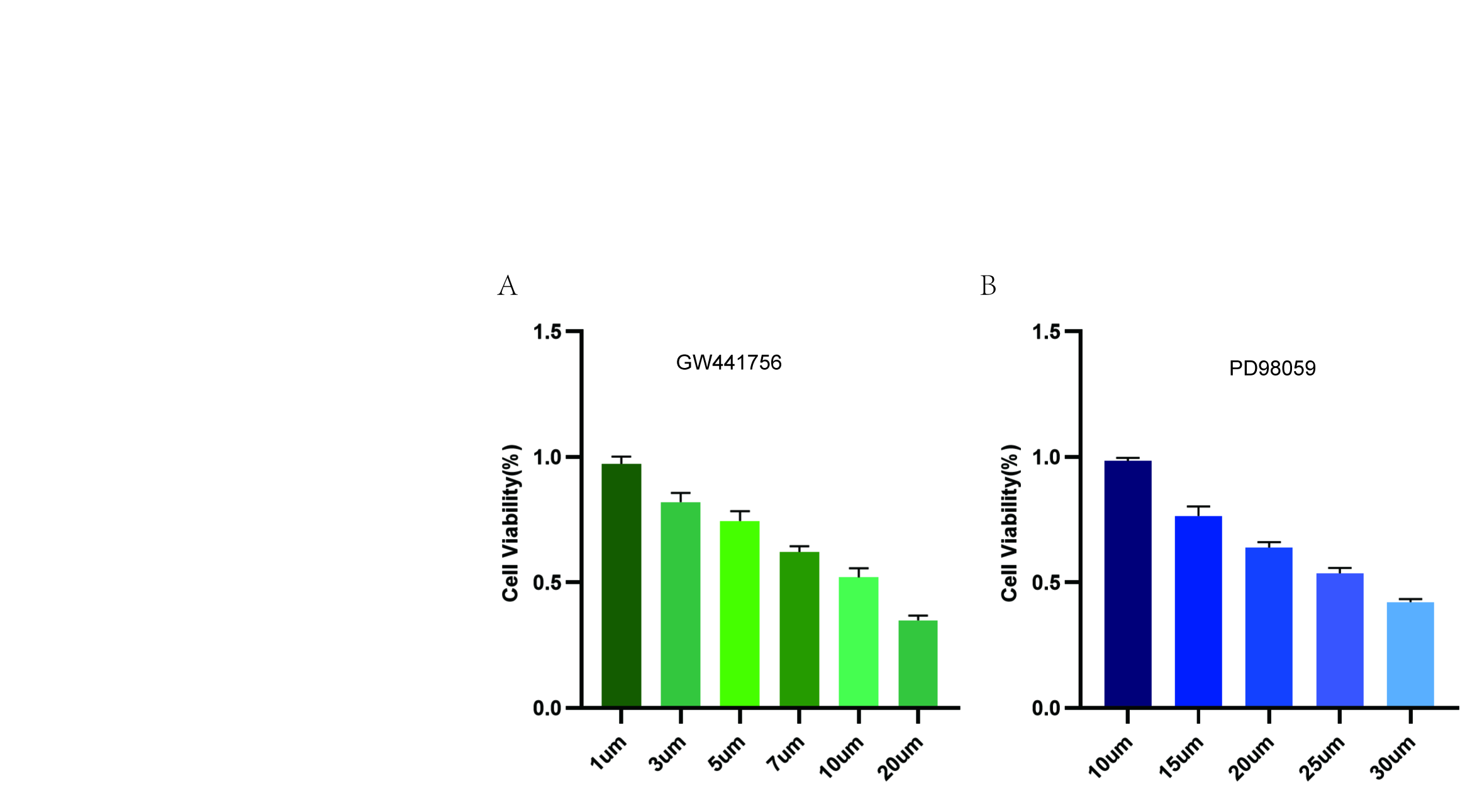

Supplement: Supplementary Figure 1 — Effect of different concentrations of GW441756 and PD98059 on cell viability.Cell viability was assessed using the CCK-8 assay following treatment with various concentrations of (A) GW441756 and (B) PD98059. [file Image1.tif]
